# Studies on sand fly fauna and ecological analysis of *Phlebotomus orientalis* in the highland and lowland foci of kala-azar in northwestern Ethiopia

**DOI:** 10.1371/journal.pone.0175308

**Published:** 2017-04-06

**Authors:** Esayas Aklilu, Araya Gebresilassie, Solomon Yared, Mizan Kindu, Habte Tekie, Meshesha Balkew, Alon Warburg, Asrat Hailu, Teshome Gebre-Michael

**Affiliations:** 1 Department of Biology, Mada Walabu University, Bale-Robe, Ethiopia; 2 Department of Biology, Jigjiga University, Jigjiga, Ethiopia; 3 Department of Zoological Sciences, Addis Ababa University, Addis Ababa, Ethiopia; 4 Aklilu Lemma Institutes of Pathobiology, Addis Ababa University, Addis Ababa, Ethiopia; 5 Department of Microbiology and Molecular Genetics, The Institute of Medical Research Israel-Canada The Kuvin Center for the Study of Infectious and Tropical Diseases, Faculty of Medicine, The Hebrew University, Hadassah Medical School, Jerusalem, Israel; 6 Department of Microbiology, Immunology and Parasitology, College of Health Sciences, Addis Ababa University, Addis Ababa, Ethiopia; University of Minnesota, UNITED STATES

## Abstract

**Background:**

Visceral leishmaniasis (VL) also known as kala-azar is a growing health problem in Ethiopia with an estimated annual VL incidence between 3700 and 7400. The disease is mainly endemic in northwestern parts of the country. The aim of the current study was to determine the sand fly fauna and ecology of *Phlebotomus orientalis* in two endemic and ecologically distinct areas of northwestern Ethiopia.

**Methods:**

Sand flies were collected using CDC light traps, sticky traps and pyrethrum spray catches from peri-domestic, mixed forest, farm field and indoor habitats from both Libo-Kemkem (May 2011-April 2012) and Metema (October 2012-September 2013) districts.

**Results:**

A total of 51,411 sand fly specimens were collected and identified (10,776 from highland and 40, 635 from the lowland areas). Seven species were found in the highland area: two *Phlebotomus* spp. (*P*. *orientalis* and *P*. *rodhaini*) and five *Sergentomyia* species. Whereas 19 species were found in the lowland area: six *Phlebotomus* (*P*. *orientalis*, *P*. *rodhaini*, *P*. *bergeroti*, *P*. *duboscqi*, *P*. *papatasi* and *P*. *martini*) and 13 *Sergentomyia* species. Of the *Phlebotomus* spp., *P*. *orientalis* was the predominant species in both the highland (99.9%) and lowland (93.7%) areas. Indoor collections using pyrethrum spray catches and sticky traps indicated that *P*. *orientalis* has a strong exophilic and exophagic behaviors in both districts. In both areas, this species showed seasonal occurrence and showing abundance during the dry months (March-May/June) of the year and increasing in numbers till the rain season, when numbers dropped dramatically. Mean density of *P*. *orientalis* in the two areas had positive and significant correlation with mean temperature in light trap collections (P<0.05). However, mean density of *P*. *orientalis* in the two areas in sticky trap collections had positive and insignificant association with the temperature (P>0.05). Regarding the rainfall pattern, density of *P*. *orientalis* had negative and statistically insignificant correlation (for light trap collections for both areas) and significant correlation (for sticky trap collections for lowland area).

**Conclusions:**

The current study indicated the variation in sand fly fauna between the highland and lowland districts, wherein, *P*. *orientalis* was found to be the most abundant *Phlebotomus* species. The study also determined that *P*. *orientalis* exhibits distinct seasonality, where its abundance increases during the dry season and disappears when the rainy period starts in both study areas. This entomological observation on the bionomics of *P*. *orientalis* provides significant evidence for considering vector control or preventive measures in the areas studied.

## Introduction

In Ethiopia, visceral leishmaniasis (VL) or kala-azar is caused by *Leishmania donovani*, and is a growing health problem with an estimated annual incidence that ranges between 3700 and 7400 [1]. The well-known endemic areas of the disease are found in the Metema-Humera lowlands in the northwest bordering with Sudan, where it accounts for about 60% of the total cases and in arid areas of south and southwest of the country [2]. Besides, recently the disease has spread to the highlands of Libo-Kemkem and Fogera districts, where it claimed the lives of more than 200 people and nowadays the disease is a serious health concern in the districts and also neighboring areas [3, 4, 5].

*Phlebotomus orientalis* is a proven vector of *L*. *donovani* in eastern Sudan [6] and the suspected vector in the northwestern Ethiopia [7, 8]. In these areas, it is strongly associated with *Acacia-Balanites* forest and deeply cracking ‘black cotton soil’, which the species may use as breeding and resting sites [9, 10]. Previous studies on population dynamics and distribution of this species in Sudan revealed that *P*. *orientalis* is a seasonal species and abundant mainly in the drier period of year (February-June) [11, 12, 13]. However, detailed studies on the bionomics of phlebotomine sand flies in northwestern Ethiopia particularly in Libo-Kemkem and Metema districts are limited [14]. Information on the population dynamics, distribution and behaviors of vector species are very important to understand when, where and how humans are infected with *Leishmania* parasites. Furthermore, this knowledge is the basis towards developing appropriate vector control methods [15]. As the information available about sand fly vector species is scarce in northwestern Ethiopia especially in Libo-Kemkem and Metema districts, detailed studies were conducted in the highland and lowland foci of the region aimed to determine sand fly fauna and bionomics of *P*. *orientalis*.

## Material and methods

### Study areas

The study was conducted in two ecologically distinct areas of Amhara Regional State in northwestern Ethiopia, namely Libo-Kemkem and Metema districts. The former is situated in a highland and the latter in a lowland area and the distance between the two is approximately 255 kms. In both localities, kala-azar is a major public health problem.

#### Libo-Kemkem

**Fig 1:**
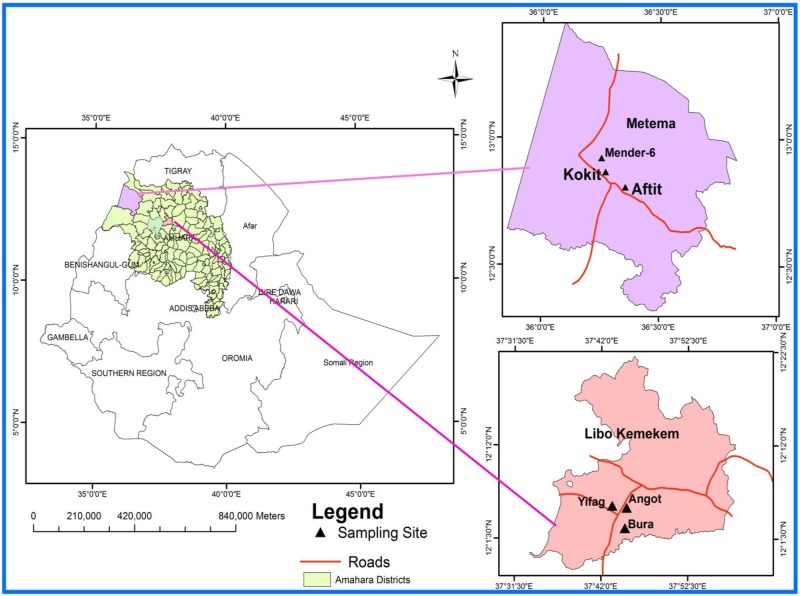
Map of the study villages in Libo-Kemkem and Metema districts northwestern Ethiopia.

In Libo-Kemkem, entomological investigation was carried out for one year from May 2011 to April 2012. The district is found about 645km northwest of Addis Ababa with a latitude and longitude of 12° 04’N and 37° 45’E, respectively. The district is also located at average altitude of 2000 meters above sea level (asl). The mean annual temperature of the area is 20.3°C. The area receives annual rainfall of 1350 mm. The majority of the soil type of the area is vertisol (black cotton soil). Most of the people live in huts constructed of mud walls and thatched roofs with some living in corrugated iron roofs. The natural vegetation coverage of the area (mostly *Acacia seyal*) has been immensely reduced mainly for agricultural purpose, construction of houses and fire wood. This natural vegetation has been replaced by *Eucalyptus* trees. Nowadays, there are a few scattered clumps of *Acacia* spp. including *A*. *seyal* in the area. Agriculture and allied activities are the most important source of subsistence for the majority of the population. They principally produce *teff*, maize, millet, bean, sunflower, rice and cotton during the main rainy season. They also raise a large number of livestock, including cattle, sheep, goats and poultry. For the entomological study, three villages (i.e. Angot, Bura and Yifag) of the district were selected mainly based on previous reports of VL cases in the district (Fig 1). The distance between these villages varies from 5 to 7 kms.

#### Metema

Field studies were also conducted in Metema district for one year (October 2012—September 2013). The district is located about 860 km northwest of Addis Ababa and 180 km southwest of Gondar city. It lies at latitude of 12° 46’ N, longitude of 36° 24’E and altitudes between 700 and 750 meters asl. The district has unimodal rainfall pattern, with a mean annual rainfall ranging between 850 and 1000 mm. The dry season of the area starts in October and extends until the end of May. During this period, the temperature usually ranges between 27°C and 32°C and the daily maximum temperature reaches 41°C in April. The majority of the people live in huts constructed of mud walls and thatched roof with some living in houses covered with iron-corrugated sheets. The characteristic vegetation of the area is wooded savannah which is alternating with grasslands. The main trees are *A*. *syeal*, *Balanites aegyptiaca*, *Zyzyphus spina-christa* and other trees and bushes. People in the area depend on agricultural activities as main source of income. Farming activities include cultivation of cash crops (such as sesame and cotton), and staple crops (*teff*, maize and sorghum). They also raise cattle, sheep and goats. For entomological study, three villages were selected. These villages were Aftit, Kokit and Mender-6 (Fig 1). The distance between these villages varies approximately from 12 km to 20 kms.

### Collection of sand flies

Adult phlebotomine sand flies were collected using three sampling techniques; CDC light traps, sticky traps and pyrethrum spray catch [16, 17].

#### CDC light traps (LTs)

In both districts, three permanent sampling habitats were selected: peri-domestic (outside human dwelling), farm field and mixed forest. The distance between the habitats ranged between 150 to 200 m. In each habitat, two LTs were deployed. During the sampling night, it was hanged 30–50 cm above the ground from dusk to dawn. A total of 710 CDC-LTs (447 in highland and 263 in lowland) were deployed. These CDC-LTs were working for 147 nights (83 nights in the highland and 64 nights in the lowland) with a total of 104, 370 (710 LTs x 147 nights) collection trap-nights were used. In the morning, trapped sand flies were brought to the field laboratory, where they were aspirated from the cage and killed using chloroform. The collected sand flies were preserved in 70% ethyl alcohol for later species identification. Sampling using LTs was carried out weekly in Libo-Kemkem (May 2011-April 2012) and bimonthly in Metema (October 2012- September 2013).

#### Sticky traps (STs)

Three sampling habitats including indoor, farm-field and mixed forest in each village were selected for the collection of sand fly specimens. For indoor collection, five ST sheets tied together on nylon string about 30 cm apart were placed vertically on the wall above sleeping location inside four huts in each village (i.e. a total of 20 STs per night). Likewise, in other sampling sites similar numbers of STs with that of indoor collection were deployed horizontally. Each morning, sand flies from STs were removed using forceps and stored in 96% ethyl alcohol in labeled vials for later species identification. A total of 7525 STs (4415 in the highland and 3110 in the lowland) were used to collect sand flies. These traps were working similar number of nights with that of LTs in both areas. The total numbers of trap-nights in both areas were 1106175.

#### Pyrethrum spray catches (PSCs)

Collection of sand flies using PSCs was carried out in one village of Libo-Kemkem (Bura) and another village in Metema (Kokit) to determine any endophilic sand flies. In each village, five huts were used. PSCs were conducted in the morning from 6:00–8:00 hrs once a month for six months (November 2011-April 2012) in Bura and seven months (December 2012-June 2013) in Kokit. For this purpose, all occupants, removable objects, exposed food and water were removed from each hut and any openings were properly closed. The entire floor was covered with white plastic sheets (4×3m), after which an aerosol insecticide (Roach-Killer-M/s Kafr Elzayat, Maybanz Plc, Egypt) was sprayed for about 5min inside the hut. After spraying, hut was closed for 15min to produce a knockdown effect. Then, the white plastic sheet was taken outside and all knocked down sand flies were collected using forceps and placed in 70% alcohol for species identification [17].

### Mounting and identification of sand flies

Sand flies collected during the study were mounted on microscope slides in Hoyer’s medium with their heads separate from thoraces and abdomens. Identification of the species was made by examining male genitalia, and the female spermathecae and pharynx according to the morphological keys of Quate [11] and Abonnenc and Minter [18]. Additional morphological keys of Lane and Fritz [19], and Gebre-Michael and Medhin [20] were also used to separate sympatric species of the subgenus *Phlebotomus* in Metema.

### Meteorological data

In order to observe the relation of weather variables with the monthly abundance of sand flies, total rainfall and mean temperature for each month for the study period of Libo-Kemkem (2011/12) and Metema (2012/13) were obtained from the National Meteorological Services Agency of Ethiopia Bahir Dar branch.

### Ethical approval

Verbal informed consent was obtained from heads of the households to collect sand flies using sticky traps and pyrethrum space spray catches inside their homes.

### Data analysis

The data were entered in SPSS sheet and statistical analysis was made with SPSS version 20.0 software (SPSS Inc, Chicago, IL, USA). Density of sand fly was calculated as number of sand fly per trap per night (LT and ST) to determine seasonality and to compare between habitats. Prior to data analysis, sand fly numbers were log-transformed [log (n+1)] to fit normal distribution and checked for normality by Shapiro-Wilk test. When trapping data did not conform to the normal distribution, the non-parametric equivalent tests of Mann Whitney U-test and Kruskal-Wallis test were used. Kruskal-Wallis test was applied to compare the mean numbers of *P*. *orientalis* collected in the three sampling habitats using CDC-LTs and STs. Whitney U-test was used to compare sex ratio in trapping methods. To estimate the relationships between weather variables and the overall density of *P*. *orientalis* per trap per night Pearson correlation coefficient (r) was used. All statistical tests were significant at P < 0.05. Ecological comparisons between the study districts and between the villages in the district were performed using the following indices:

**Shannon-Wiener index**: $\text{H'}=-\Sigma_{i=1}^{s}(\text{Pi)lnPi}$; where S is the number of species and Pi is the proportion of the total samples belonging to i-th species. H’ expresses the differences in the diversity of the sand fly fauna between villages and districts

**Evenness**: E = H’/ln(S), Where H’ is the value of Shannon-Wiener, E = expresses how evenly the individuals in the community are distributed over the different species [21].

**Species richness**: S = the number of species in each district [22]

## Results

### Species composition and relative abundance

A total of 51,411 sand fly specimens were collected during the study period from six villages of the two districts using LTs, STs and PSCs. In Libo-Kemkem, seven species of sand flies were caught, belonging two subgenera of the genus *Phlebotomus* (86.6%): *Phlebotomus* (*Larroussius*) *orientalis* and *P*. (*Anaphlebotomus*) *rodhaini* and four subgenera of *Sergentomyia* spp. (13.4%): *Sergentomyia* (*Sergentomyia*) *bedfordi* group, *S*. *(Sergentomyia*) *schewtzi*, *S*. (*Grassomyia*) *squamiplueris*, *S*. (*Parrotomyia*) *africana* and *S*. (*Sintonius*) *clydei* (Table 1). In Metema, however 19 species of sand flies (i.e. Six *Phlebotomus* and 13 *Sergentomyia)* belonging to four subgenera of *Phlebotomus*: *P*. *orientalis*, *P*. *(Phlebotomus) bergeroti*, *P*. (*Phlebotomus*) *papatasi*, *P*. (*Phlebotomus*) *duboscqi*, *P*. *rodhaini* and *P*. (*Synphlebotomus*) *martini* and. four subgenera of *Sergentomyia* (*Sergentomyia*, *Parrotomyia*, *Grassomyia* and *Sintonius*), which constituted 88.3% of the total collection (Table 1).

**Table 1:** Species composition and relative abundance of phlebotomine sand flies collected by various methods in Libo-Kemkem (May 2011-April 2012) and Metema (October 2012-September 2013) districts in northwest Ethiopia.

Species	Type of collections												Overall Total (%)			
	Light trap				Sticky trap				Pyrethrum spray catch							
	Libo-Kemkem		Metema		Libo-Kemkem		Metema		Libo-Kemkem		Metema		Libo-Kemkem		Metema	
	M/F	Total (%)	M/F	Total(%)	M/F	Total (%)	M/F	Total (%)	M/F	Total(%)	M/F	Total (%)	M/F	Total(%)	M/F	Total(%)
*P*.*orientalis*	4154/3159	7313(87.8)	1729/1239	2967(9.4)	1925/91	2016(82.2)	1375/90	1466(16.6)	-	-	-/2	2(0.6)	6079/3250	9329(86.6)	3104/1331	4435(10.9)
*P*. *rodhaini*	-/1	1(0.01)	52/147	199(0.63)	-	-	22/30	52(0.6)	-	-	-	-	-/1	1(0.001)	74/177	251(0.6)
*P*.*duboscqi*	-	-	5/3	8(0.025)	-	-	-	-	-	-	-	-	-	-	5/3	8(0.02)
*P*.*papatasi*	-	-	2/0	2(0.006)	-	-	1/0	1(0.01)	-	-	-	-	-	-	3/0	3(0.007)
*P*.*bergeroti*	-	-	26/4	30(0.095)	-	-	-	-	-	-	-	-	-	-	26/4	30(0.74)
*P*. *martini*	-	-	1/1	2(0.006)	-	-	-	-	-	-	-	-	-	-	1/1	2(0.005)
*S*. *clydei*	2/5	7(0.08)	2914/6164	9078(28.8)	-	-	982/1152	2134(24.2)	-	-	2/2	4(1.2)	2/5	7(0.001)	3898/7318	11216(27.6)
*S*. *africana*	14/31	45(0.5)	2267/1092	3359(10.7)	20/8	28 (1.1)	462/357	819(9.3)	-	-	22/54	76(23.9)	34/39	73 (0.68)	/1503	4254(10.5)
*S*. *bedfordi*	316/176	492(5.9)	2731/3840	6571(20.87)	138/65	203(8.2)	360/355	715(8.1)	-	-	2/14	16(5.04)	454/241	695(6.5)	3093/4209	7302(18.0)
*S*.*schewtzi*	161/4	165(1.98)	564/1148	1712(5.4)	50/3	53 (2.2)	537/680	1217(13.8)	-	-	-	-	211/7	218(2.02)	1101/1828	2929(7.3)
*S*.*squamiplueris*	144/154	298 (3.73)	1702/2179	3881(12.3)	56/99	155 (6.3)	909/1097	2006(22.7)	-	-	1/1	2(0.6)	200/253	453(4.2)	2612/3277	5889(14.6)
*S*. *adleri*	-	-	40/24	64(0.2)	-	-	26/1	27(0.3)	-	-	-	-	-	-	66/25	91(0.22)
*S*. *antennata*	-	-	2666/911	3577(11.4)	-	-	178/207	385(4.4)	-	-	13/203	216(68.1)	-	-	2857/1321	4178(10.3)
*S*. *yusafi*	-	-	4/0	4(0.012)	-	-	3/0	3(0.03)	-	-	-	-	-		7/0	7(0.017)
*S*. *affinis*	-	-	2/0	2(0.006)	-	-	-	-	-	-	-	-	-	-	2/0	2(0.005)
*S*. *buxtoni*	-	-	2/1	3(0.009)	-	-	-	-	-	-	-	-	-	-	2/1	3(0.074)
*S*. *dubia*	-	-	1/27	28(0.08)	-	-	0/4	4 (0.05)	-	-	-	-	-	-	1/31	32(0.08)
*S*. *suberecta*	-	-	1/1	2(0.006)	-	-	-	-	-	-	-	-	-	-	1/1	2(0.005)
*S*. *christophersi*	-	-	-	-	-	-	-	-	-	-	0/1	1(0.3)	-	-	0/1	1(0.005)
**Total**	**4791/3530**	**8321(77.2)**	**14709/16781**	**31490(77.5)**	**2189/266**	**2455(22.8)**	**4855/3973**	**8828(21.7)**	**-**	**-**	**40/277**	**317(0.8)**	**6980/3796**	**10,776**	**19605/21030**	40635

The relative abundances of sand fly species in the highland and lowland areas are depicted in Table 1. In Libo-Kemkem, *P*. *orientalis* was the most prevalent species which constituted 99.9% and 86.6% of *Phlebotomus* spp. and the total sand fly specimens collected, respectively. While the remaining species in descending order were *S*. *bedfordi* (6.5%), *S*. *squamiplueris* (4.2%), *S*. *schewtzi* (2.02%), *S*. *africana* (0.68%), *S*. *clydei* (0.001%) and *P*. *rodhaini* (0.0001%). Among the *Phlebotomus* spp. in Metema, *P*. *orientalis* was the most preponderate species (93.8%) followed by *P*. *rodhaini* (5.3%), *P*. *bergeroti* (0.6%), *P*. *duboscqi* (0.2%), *P*. *papatasi* (0.06%) and *P*. *martini* (0.04%). Of all sand fly species in the lowland area, *S*. *clydei* was the most abundant species accounting for 27.6% of the total collection, and followed by *S*. *bedfordi* (18.0%), *S*. *squamipleuris* (14.6%), *P*. *orientalis* (10.9%), *S*. *africana* (10.5%), *S*. *antennata* (10.3%) and *S*. *schewtzi* (7.3%).

### Indoor resting habits (endophilic behavior) of phlebotomine sand flies

Sampling of endophilic phlebotomine sand flies in Libo-Kemkem using PSCs was not productive in collecting single sand flies, whereas in Metema, 317 phlebotomine sand flies were collected. The most endophilic sand fly was *S*. *antennata* (68.1%) followed by *S*. *africana* (23.9%) and *S*. *bedfordi* (5.0%). Only two (0.06%) females of *P*. *orientalis* were found resting indoor (Table 1).

### Sex ratio

In Libo-Kemkem, sex ratios (males: females) for different sand fly species demonstrated that males caught by all methods were higher than that of females (6980 male: 3796 female), with an overall sex ratio of 1.84:1. For *P*. *orientalis*, the sex ratio in LTs was 1.3:1, which did not show any significant difference between sexes (Mann Whitney U-test, P>0.05). Although there was no statistical difference, the sex ratio of male to female (21.2:1) in the STs was clearly higher than that of LTs collection. Unlike Libo-Kemkem, the sex ratio in Metema for different sand fly species was female biased in all collection methods (19604 males: 21031 females), with an overall sex ratio of 0.93:1. For *P*. *orientalis*, the sex ratio in LTs was 1.4:1, which did not show any significant difference between sexes (Mann Whitney U-test, P>0.05) as opposed to a very high ratio of male to female (15.3:1) in the STs that was clearly significant (P = 0.014).

### Sand fly diversity between districts and between villages

There was a difference in the diversity of the sand fly fauna between the two districts as indicated by the values of Shannon-Weiner index (H’). The H’ for Metema (1.95) was higher than that of Libo-Kemkem (0.55). Equally, the richness (S) and evenness (E) of sand fly fauna were maximal at the low altitude district (Table 2).

**Table 2:** The Shannon-Weiner diversity index (H’), evenness (E) and richness (S) of the sand fly species from the study areas.

District and villages	H’	E	S
**Libo-Kemkem**	**0.55**	**0.28**	**7**
**Angot**	1.19	0.66	6
**Bura**	0.23	0.12	7
**Yifag**	0.58	0.42	4
**Metema**	**1.95**	**0.66**	**19**
**Aftit**	1.59	0.64	12
**Kokit**	1.82	0.66	17
**Mender-6**	1.81	0.63	17

### Population dynamics of *P*. *orientalis* in the highland and lowland areas

**Fig 2:**
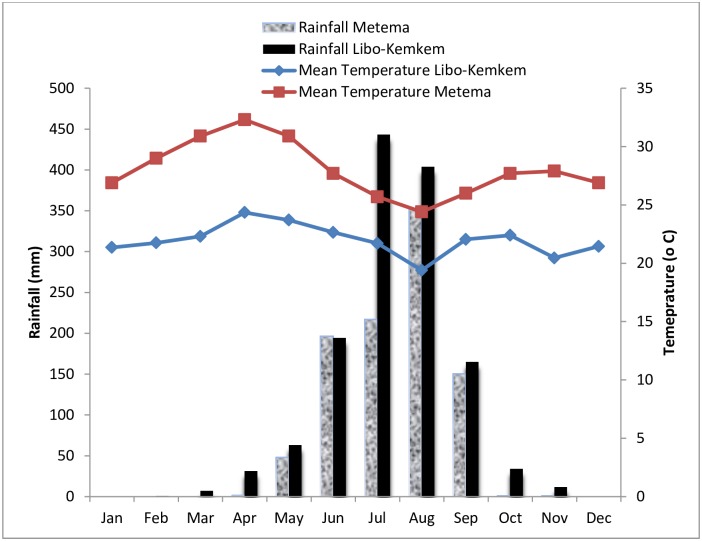
Monthly total rainfall and mean temperature of Libo-Kemkem (May 2011-April 2012) and Metema (October 2012- September 2013) districts in northwest Ethiopia.

During the dry season (October-May) in Libo-Kemkem, monthly maximum temperature of the study area ranged from 31.6 to 35°C while monthly minimum temperature varied from 9.3 to 14°C. The total monthly rainfall in this period ranged from 0 to 33.9 mm (Fig 2). Whereas in Metema during this period, monthly maximum temperature of the area ranged from 36.3°C to 41.3°C while monthly minimum temperature was in the range of 16.3°C to 23.4°C. The total monthly rainfall in this period ranged between 0 and 47.8mm (Fig 2). In wet season (June-September) in Libo-Kemkem, mean monthly maximum temperature ranged from 25.5 to 29.6°C and mean monthly minimum temperature varied from 13.3°C to 15.7°C. The total monthly rainfall during this time ranged from 165 to 443.3mm (Fig 2). Whereas in Metema during wet season, monthly maximum temperature ranged from 29.4°C to 34.7°C and monthly minimum temperature varied between 19.3°C and 20.6°C. The monthly rainfall during this time ranged from 150.4mm to 352.7mm (Fig 2).

**Fig 3:**
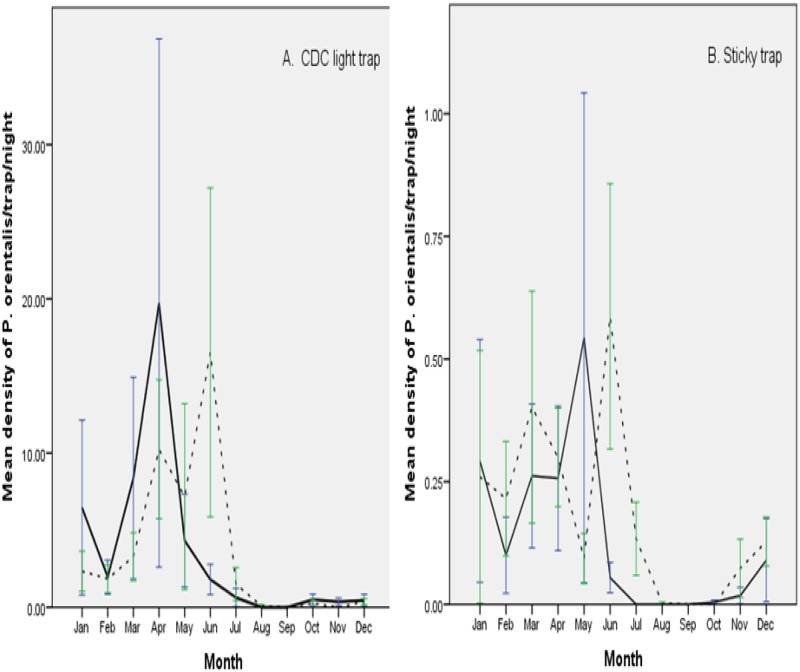
Seasonal pattern of *P*. *orientalis* in Libo-Kemkem (May 2011—April 2012) and Metema (October 2012 –September 2013) districts collected by light and sticky traps in northwest Ethiopia, N.B. Solid line–Libo-Kemkem; Broken line- Metema.

The seasonal fluctuation of *P*. *orientalis* collected by LTs and STs pooled from the three villages of each district is depicted in Fig 3A and 3B, respectively and S1 Fig. In Libo-Kemkem, there was a distinct seasonal fluctuation in the abundance of *P*. *orientalis* over 12 months of collection periods, showing an overall increased in density between January and May, with its highest peak of abundance in April (19.7± 17.1 flies/trap/night) for LTs and May (0.54±0.49 flies/trap/night) for STs. The mean temperature during these months was 24.0°C. However, *P*. *orientalis* population density drastically declined during the peak rainy season (July-September) with complete disappearance between August and September. Similar to Libo-Kemkem, the activity of *P*. *orientalis* in Metema was in sharp decline or completely absent during the rainy season (July-September) and only present in the dry season (November-June) with greater abundance towards the end of the dry season. The peak density of this species was observed to be June for both trapping methods (16.5±10.7 flies/LT/night and 0.58±0.27 flies/ST/night) when the average temperature was also high (27.7°C).

To assess the impact of weather variables on mean density of *P*. *orientalis*, correlation analysis was made for both collection methods. In Libo-Kemkem, mean density of *P*. *orientalis* had a significant positive correlation with mean temperature (r = 0.671, P = 0.017) and a non-significant negative association with the rainfall (r = -0.452, P>0.05) in LT collections. Mean monthly density of *P*. *orientalis* in STs was positively correlated with the mean temperature (r = 0.430), though the interaction was not significant (P>0.05) and negatively correlated with the rainfall and also statistically significant (r = -0.627, P<0.05). Similar to the highland, density of *P*. *orientalis* had statistically significant positive correlation with the mean temperature (r = 0.639, P<0.05), and negatively correlated with rainfall in LT collections. Furthermore, in ST collections, the correlation analysis showed that the density of *P*. *orientalis* was positively associated with the mean temperature and, negatively associated with rainfall even though not significant (P>0.05).

### Habitat preferences of *P*. *orientalis* in Libo-Kemkem and Metema

In highland, significant difference was recorded in mean density of *P*. *orientalis* among the three sampling habitats using STs (χ^2^ = 6.174, df = 2, P = 0.046) (Table 3, S1 Table). *Phlebotomus orientalis* was significantly abundant in farm fields (0.093±.03201flies/ST/night) than mixed forest (0.026±0.015flies/ST/night) or indoor (0.002±.001 flies/ST/night). Similarly, significant difference was observed in mean density of *P*. *orientalis* males among the three habitats with similar trend as that of the two sexes combined (χ^2^ = 6.05, df = 2, P<0.05). However, with females a non-significant similar trend in habitat preferences was noted (P>0.05). In contrast, there was no significant difference among the three sampled habitats (peri-domestic, farm field and mixed forest) in density of *P*. *orientalis* observed using LTs (P>0.05) (Table 4, S2 Table). In the lowland, similar significant differences in habitat preferences of *P*. *orientalis* collected by STs was obtained as in Libo-Kemkem (χ^2^ = 5.9, df = 2, P = 0.05) (Table 3). Mean density of *P*. *orientalis* was more abundant in farm fields (0.177±0.03 flies/ST/night), followed by mixed forest (0.0862±0.03 flies/ST/night) and indoors (0.07±0.02 flies/ST/ night). When habitat preferences of *P*. *orientalis* was analyzed in terms of sex; males of *P*. *orientalis* were markedly abundant (P = 0.04) in farm fields (0.112±0.03flies/ST/night) than mixed forest (0.08±0.034flies/ST/night) and indoor (0.006±0.002flies/ST/night). On the other hand, for females a non-significant similar trend in habitat preferences was observed (P>0.05). In contrast to the ST capturing, mean density of *P*. *orientalis* collected by LTs was higher in mixed forest (2.88±1.6 flies/LT/ night) and lower in abundant in peri-domestic (1.01±0.49 flies/LT/ night), though it was not significant (P>0.05) (Table 4).

**Table 3:** Mean density (±SE) of *P*. *orientalis* collected per sticky trap/night from different sampling habitats in Libo-Kemkem (May 2011 to April 2012) and Metema (October 2012 to September 2013).

Habitat	District					
	Libo-Kemkem			Metema		
	Total no. males (mean/ST/night ± SE)	Total no. females (mean/ST/night ± SE)	Total no. (mean/ ST/night ± SE)	Total no. males (mean/ST/night ± SE)	Total no. females (mean/ST/night ± SE)	Total no. (mean/ ST/night ± SE)
**Indoor**	0.001±.001^a^	0.0004±0.0002^a^	0.002±.001^a^	0.006±0.002^a^	0.001±0.0005^a^	0.006±0.002^a^
**Farm field**	0.089±0.031^b^	0.004±0.001^b^	0.093±.032^b^	0.112±0.029^b^	0.006±0.0015^b^	0.117±0.0306^b^
**Mixed forest**	0.024±0.014^ab^	0.002±0.001^ab^	0.026±.0145^ab^	0.08±0.034^ab^	0.006±.0022^ab^	0.086±0.0352^ab^

Mean values followed by different letters in the same column are statistically significant (Kruskal-Wallis test, P < 0.05)

**Table 4:** Mean density (± SE) of *P*. *orientalis* collected in CDC light traps/night from different sampling habitats in Libo-Kemkem (May 2011 to April 2012) and Metema (October 2012 to September 2013).

Habitat	District					
	Libo-Kemkem			Metema		
	Total no. males (mean/CDC/night ± SE)	Total no. females (mean/CDC/night ± SE)	Total no. (mean/ CDC/night ± SE)	Total no. males (mean/CDC/night ± SE)	Total no. females (mean/CDC/night ± SE)	Total no. (mean/ CDC/night ± SE)
**Peri-domestic**	0.696±0.342^a^	0.582±0.310^a^	1.2784±.63614^a^	0.537±0.246^b^	0.472±0.256^b^	1.010±0.492^b^
**Farm field**	0.809±0.399^a^	0.690±0.259^a^	1.4993±.64831^a^	0.644±0.191^b^	0.418±0.130^b^	1.064±0.298^b^
**Mixed forest**	0.087±.0209^a^	0.059±0.017^a^	0.1468±0.03016^a^	1.74±0.955^b^	1.138±0.766^b^	2.887±1.685^b^

Mean values followed by the same letters in the same column are statistically not significant (Kruskal-Wallis test, P > 0.05).

## Discussion

In the present study, more than fifty thousand phlebotomine sand flies of two genera (*Sergentomyia* and *Phlebotomus*) were trapped from the highland (Libo-Kemkem) and the lowland (Metema) districts in northwest Ethiopia. There was a significant difference in the diversity, evenness and richness of sand fly fauna between the two sites, indicating the patchy distributions of sand fly species in nature across altitudinal gradient. This distribution difference could be the result of variation in elevation between the two sites, which, in turn, creates variation in biotic and abiotic factors [23]. Guernaoui et al. [24] in Morocco noted high species richness and diversity at low altitude as compared to high altitude.

Seven species of sand fly *(P*. *orientalis*, *P*. *rodhaini*, *S*. *bedfordi*, *S*. *squamiplueris*, *S*. *schewtzi*, *S*. *africana* and *S*. *clydei*) were encountered in the highland district. Of these, *P*. *orientalis* was the predominant species and is the suspected vector of *L*. *donovani* in the area and in northwest Ethiopia [4,8] and the proven vector of the parasite in neighboring Sudan [6, 25]. Despite some variations in the type of sand fly species recorded in the present study, the overall species composition is in agreement with Gebre-Michael et al. [4].

In addition to the above seven species of sand flies, another 13 species were recorded in Metema, lowland district. Unlike Libo-Kemkem, the majority of collected and identified species in this district belong to the genus *Sergentomyia* (88.9%). Such dominance of *Sergentomyia* spp. over *Phlebotomus* spp. in the lowland area could be due to in the lowland area there are a number of breeding habitats which support the different species of this genus [26]. The predominance of *Sergentomyia* spp. over *Phlebotomus* spp. was also previously reported in various parts of Ethiopia [27, 28] and also in neighboring Sudan, which have similar ecology and climatic condition with the present study area (Metema) [13, 29].

The genus *Phlebotomus* in Metema district consists of six species, namely *P*. *orientalis*, *P*. *bergeroti*, *P*. *papatasi*, *P*. *duboscqi*, *P*. *rodhaini* and *P*. *martini*. Like Libo-Kemkem, *P*. *orientalis* is the prevalent species and the first five species are previously recorded in the area [8]. However, *P*. *martini*, the proven vector of *L*. *donovani* in southern Ethiopia [27] and Kenya [30], is the first record for the area.

The absence of the three species of the subgenus *Phlebotomus* (*P*. *bergeroti*, *P*. *papatasi*, and *P*. *duboscqi*) in the highland site might be due to the members of the subgenus preferring areas with high ambient temperature [19, 31] The role of these sympatric species in the epidemiology of leishmaniasis in the lowland area is unclear, although recently a man had been admitted and treated for cutaneous leishmaniasis (CL) at Metema hospital (unpublished data). Elsewhere these species are either suspected or proven vectors of zoonotic CL. For instance, *P*. *papatasi* is a proven vector for *L*. *major* throughout North Africa, Middle East, Central and Eastern Asia [32], whereas *P*. *duboscqi* has also a similar role in the Sahel region of Africa [32]. This species was also reported to be a vector of *L*. *major* in the southern VL foci of Ethiopia [33].

Sampling to determine the indoor resting habits of sand fly species in general and *P*. *orientalis* in particular using pyrethrum spray catches failed to collect any sand fly specimen in the highland while few specimens of *P*. *orientalis* in the lowland area were collected. This observation implied that *P*. *orientalis* in both areas has a propensity of exophilic (resting outdoor) behavior and it is in agreement with those of earlier studies in Sudan [12, 13] and neighboring districts in northern Ethiopia [28]. Furthermore, from epidemiological point of view, this observation will have an important implication for control of VL based using vector control tools. Conventional vector control methods, such as indoor residual spraying (IRS), which is currently used for the control of the vector of kala-azar vector in India [34] and also malaria vectors in Africa, including Ethiopia [35] may not have a significant impact on the density of *P*. *orientalis* in these districts.

It is noteworthy to mention the low abundance of this sand fly species inside human dwelling (indoor) collected by non-attractive STs in both study areas. The result indicated that this species is principally an outdoor species wherever it occurs as has previously been shown by several investigators [8, 12, 13], but in disagreement with Lambert et al. [29] where 79% of the total collected *P*. *orientalis* by LTs were from inside human dwellings in eastern Sudan. This difference may be due to variation in the type of trapping methods used as LTs lure phototropic exophagic flies from outside human dwelling to enter inside [36]. This observation in both districts has significance in planning control measures against VL.

From the results obtained in this study, it was clearly shown that most sand flies, including *P*. *orientalis*, was virtually absent during the rainy season and abundant in the dry season (November-May/June). Dry season occurrence and abundance of *P*. *orientalis* has previously also noted by several investigators in Sudan [12, 13] and in Ethiopia [14, 28], although peak months of abundance may vary with locality and method of collection. Thus, in the highland area, *P*. *orientalis* was mainly active during the dry season (October-April) of the year (showed two distinct population peaks between the trapping methods, the highest density being in April (LTs) and May (STs)). Similarly, in the lowland area the species activity was maximal in the low precipitation period with population peak in June for both trapping methods. Such difference in population peaks between the two areas may be due to climatic differences, e.g. the highland area is cooler and more humid during the dry season than the lowland area.

In the lowland area, migrant workers from the surrounding highland regions (Gondar, Gojam and Tigray) make their first arrivals usually at the end of May. This migration is seasonal, and coincides with the different agricultural activities such as clearing of the natural habitat/forest for preparing a land and subsequent weeding and harvesting. This period also concurs with the peak abundance of *P*. *orientalis* in May and June (just before the rainy season) in the area, leading the migrant workers to be exposed to biting challenges of *P*. *orientalis* and increased chances of being infected with *L*. *donovani*. This prevailing situation could also exacerbate an outdoor transmission of *L*. *donovani* in this particular area where most of the migrant laborers sleep outdoors nearby farmlands in the natural habitats without any protection such as bed net [Pers. Observation].

In the current study, temperature showed positive significant correlation with monthly density of *P*. *orientalis*, which is in agreement with earlier observation in Sudan [13]. In both areas, density of *P*. *orientalis* reached peak before the onset of the main rainy season when the mean temperature was <30°C (24.4°C for highland area and 27.7°C for lowland area). However, rainfall had negative correlation with the densities of the species in both areas. The negative association between the density of the species and rainfall in the present study corroborated previous works by Quate [11] and Hoogstraal and Heyneman [12] as all of them could not find a single adult *P*. *orientalis* during the rainy period in Sudan. Such indirect association was also pointed out by Gebre-Michael et al. [9]. A similar negative association between rainfall patterns and abundance of *P*. *orientalis* was recorded in the district of Tahtay Adiyabo, northern Ethiopia [28].

Habitat preference study of *P*. *orientalis* collected by STs in both districts showed that farm field was the most productive habitat than that of mixed forest and indoor habitats. Similar findings have been observed by Lemma et al. [14] in Kafta-Humera district, north of Metema district. In both areas, majority of the farm field consists of black-cotton soil. Previously, it was indicated that the distribution of *P*. *orientalis* was largely associated with such soil type in East Africa [9, 37]. Such type of soil is characterized by high contents of clay minerals that enhance swelling when hydrated and shrinkage upon desiccation, thereby, causing extensive cracking during the dry season [10]. Furthermore, a study conducted in Humera area by Shabtai et al. [38] indicated that vertisols from farm fields have high swelling capacity upon wetting than vertisol in a nearby forest. Accordingly, cracks in farm fields were markedly wider and deeper than cracks in forest habitat. Such wider and deeper cracks have high humidity and stable temperature, which could provide suitable breeding and resting sites for *P*. *orientalis* [10]. However, very little is known about how *P*. *orientalis* survives the rainy season, though it may be by diapausing as late instar larvae as observed in laboratory colonies [39] or Palaearctic species or as eggs as in warmer and wetter habitats [40].

In conclusion, the current study indicated the variation in sand fly fauna between the highland and lowland districts, wherein, *P*. *orientalis* was found to be the most abundant *Phlebotomus* species. The study also indicated that *P*. *orientalis* exhibits distinct seasonality, where its abundance increases during the dry season and disappears when the rainy period starts in both study areas. Furthermore, this study reveals the exophilic and behavior of *P*. *orientalis*. This behavior has practical implication because this behavior actually makes the species less susceptible to vector control methods which are implemented in indoor conditions. In overall, this entomological observation on the bionomics of *P*. *orientalis* is significant for considering vector control or preventive measures in the areas based on ecological knowledge of VL vector.

## Supporting information

S1 Fig Population dynamics of *P*. *orientalis* in the highland and lowland areas. (PDF)

S1 Table Habitat preferences using sticky traps. (PDF)

S2 Table Habitat preferences using light traps. (PDF)
