# Supplementary material for: Studies on sand fly fauna and ecological analysis of Phlebotomus orientalis in the highland and lowland foci of kala-azar in northwestern Ethiopia
Source: PLoS One. 2017 Apr 6;12(4):e0175308. doi: 10.1371/journal.pone.0175308 (PMC5383282; doi:10.1371/journal.pone.0175308)
Supplement: S1 Fig — (PDF) [file pone.0175308.s001.pdf]

Month	desnity of P. orientalis collected using LTs in Libo	density of P. orientalis collected Using LTs in Metema	density of P. orientalis collected using STs in Lbo	density of P. orientalis collected in Metam using STs
1	1.63	0.06	0.09	0
1	17.76	2.4	0.78	0.78
1	0	4.56	0	0
2	2.07	0.17	0.05	0.03
2	3.8	3.27	0.25	0.19
2	0	2.11	0	0.43
3	3.86	0.65	0.28	0.25
3	21.27	6.04	0.51	0.87
3	0	3.17	0	0.09
4	5.33	3	0.26	0.24
4	53.85	18.5	0.51	0.5
4	0	9.25	0	0.17
5	2.88	0.8	0.09	0.11
5	0	19.23	1.54	0.18
5	10.1	1.5	0	0
6	2.02	4.3	0.06	0.39
6	3.39	37.79	0.11	1.12
6	0	7.5	0	0.25
7	0.06	0	0	0.01
7	1.83	3.58	0	0.27
7	0	0.92	0	0.12
8	0	0	0	0
8	0	0.25	0	0.01
8	0	0	0	0
9	0	0	0	0
9	0	0	0	0
9	0	0	0	0
10	0.22	0.75	0.01	0
10	1.21	0	0	0
10	0.06	0	0	0
11	0.22	0	0	0
11	0.85	0	0.05	0.19
11	0	0	0	0.03
12	0.12	0.04	0.01	0.14
12	1.23	0.3	0.26	0.21

12	0	0.75	0	0.04

N.B. 1-12= January-December
