# Supplementary material for: Studies on sand fly fauna and ecological analysis of Phlebotomus orientalis in the highland and lowland foci of kala-azar in northwestern Ethiopia
Source: PLoS One. 2017 Apr 6;12(4):e0175308. doi: 10.1371/journal.pone.0175308 (PMC5383282; doi:10.1371/journal.pone.0175308)
Supplement: S1 Table — (PDF) [file pone.0175308.s002.pdf]

Habitat	Male density Libo	Female Density Libo	Total density Libo	Male density Metema	Female density Metema	Total density Metema
1	0.13	0	0.13	0	0	0
1	0.04	0	0.04	0.06	0.01	0.07
1	0	0	0	0.1	0.01	0.11
1	0	0	0	0.28	0.01	0.28
1	0	0	0	0.2	0	0.2
1	0	0	0	0.25	0.01	0.26
1	0.02	0	0.02	0.16	0.02	0.18
1	0.05	0.01	0.06	0.08	0.01	0.08
1	0.22	0.01	0.23	0.21	0.01	0.22
1	0.1	0.01	0.11	0.01	0	0.01
1	0.34	0.01	0.35	0	0	0
1	0.17	0	0.18	0	0	0
2	0.16	0.01	0.17	0	0	0
2	0.02	0	0.02	0	0	0
2	0	0	0	0	0	0.01
2	0	0	0	0.09	0	0.09
2	0	0	0	0.02	0.02	0.04
2	0	0	0.01	0.24	0.02	0.26
2	0	0	0	0.1	0	0.1
2	0	0	0	0.03	0.01	0.04
2	0.01	0	0.01	0.38	0.01	0.39
2	0.01	0	0.01	0.1	0.01	0.11
2	0.01	0	0.01	0	0	0
2	0.08	0	0.08	0	0	0
3	0	0	0	0	0	0
3	0.01	0	0.01	0	0	0
3	0	0	0	0	0	0
3	0	0	0	0	0	0
3	0	0	0	0	0	0
3	0	0	0	0	0	0
3	0	0	0	0.02	0	0.02
3	0	0	0	0.01	0	0.01
3	0	0	0	0.01	0	0.01
3	0	0	0	0.02	0	0.02
3	0	0	0	0	0	0
3	0	0	0	0	0	0

N.B. 1= farm field;2= mixed forest; 3= indoor
