# Supplementary material for: Studies on sand fly fauna and ecological analysis of Phlebotomus orientalis in the highland and lowland foci of kala-azar in northwestern Ethiopia
Source: PLoS One. 2017 Apr 6;12(4):e0175308. doi: 10.1371/journal.pone.0175308 (PMC5383282; doi:10.1371/journal.pone.0175308)
Supplement: S2 Table — (PDF) [file pone.0175308.s003.pdf]

Habitat	Male density in Metema	Female density Metema	Total density Metema	Male density Libo	Female density Libo	Total density Libo
1	0.38	0	0.38	1.53	1.84	3.38
1	0	0	0	0.37	0.42	0.79
1	0.2	0.06	0.27	0.06	0.11	0.17
1	1.54	0.37	1.91	0	0	0
1	1.04	0.65	1.69	0	0	0
1	1.44	0.86	2.3	0.1	0.33	0.43
1	1.75	1.17	2.92	0.05	0.06	0.11
1	0.2	1	1.2	0.1	0.06	0.16
1	1	0.83	1.83	0.89	1.07	1.96
1	0.14	0.08	0.22	0.51	0.24	0.76
1	0.06	0	0.06	1.23	1.31	2.54
1	0	0	0	4.88	2.82	7.7
2	0	0	0	0.25	0	0.25
2	0	0	0	0.1	0.08	0.17
2	0	0	0	0.05	0.05	0.1
2	0.04	0	0.04	0	0	0
2	0.21	0.24	0.45	0	0	0
2	0.58	0.2	0.78	0	0.05	0.05
2	3.02	1.93	4.95	0.08	0.04	0.12
2	6.64	1.82	8.46	0.07	0.06	0.13
2	10.17	9.27	19.44	0.13	0.23	0.37
2	0.33	0.17	0.5	0.12	0.07	0.19
2	0	0.03	0.03	0.12	0.05	0.17
2	0	0	0	0.14	0.06	0.21
3	0	0	0	0.44	0.06	0.5
3	0	0	0	0.72	0.36	1.08
3	0	0.02	0.02	0	0.02	0.02
3	0.04	0.02	0.07	0	0	0
3	0.02	0.02	0.03	0	0	0
3	0.25	0.27	0.52	0	0.03	0.03
3	1.94	1.07	3.01	0.01	0.01	0.02
3	2.34	3.03	5.38	0.06	0.06	0.13
3	1.38	0.95	2.32	0.46	0.89	1.35
3	0.47	0.31	0.78	0.31	0.27	0.58
3	0	0	0	3.24	1.7	4.93
3	0	0	0	3.13	3.59	6.72

N.B. 1=Farm field; 2=Mixed forest ; 3= peri-domestic
